# Outcomes and Impact of Pre-ECMO Clinical Course in Severe COVID-19-Related ARDS Treated with VV-ECMO: Data from an Italian Referral ECMO Center

**DOI:** 10.3390/jcm13123545

**Published:** 2024-06-17

**Authors:** Gabriele Sales, Giorgia Montrucchio, Valentina Sanna, Francesca Collino, Vito Fanelli, Claudia Filippini, Umberto Simonetti, Chiara Bonetto, Monica Morscio, Ivo Verderosa, Rosario Urbino, Luca Brazzi

**Affiliations:** 1 Department of Surgical Sciences, University of Turin, 10124 Turin, Italy; giorgiagiuseppina.montrucchio@unito.it (G.M.); luca.brazzi@unito.it (L.B.); 2 Department of Anesthesia, Intensive Care and Emergency, “Città Della Salute e Della Scienza” Hospital, 10126 Turin, Italy

**Keywords:** COVID-19, SARS-CoV-2 infection, acute respiratory distress syndrome, mechanical ventilation, extracorporeal membrane oxygenation, respiratory mechanics, patient outcome assessment, hospital mortality

## Abstract

**Background:** The efficacy of veno-venous extracorporeal membrane oxygenation (VV-ECMO) as rescue therapy for refractory COVID-19-related ARDS (C-ARDS) is still debated. We describe the cohort of C-ARDS patients treated with VV-ECMO at our ECMO center, focusing on factors that may affect in-hospital mortality and describing the time course of lung mechanics to assess prognosis. **Methods:** We performed a prospective observational study in the intensive care unit at the “Città della Salute e della Scienza” University Hospital in Turin, Italy, between March 2020 and December 2021. Indications and management of ECMO followed the Extracorporeal Life Support Organization (ELSO) guidelines. **Results:** The 60-day in-hospital mortality was particularly high (85.4%). Non-survivor patients were more frequently treated with non-invasive ventilatory support and steroids before ECMO (95.1% vs. 57.1%, *p* = 0.018 and 73.2% vs. 28.6%, *p* = 0.033, respectively), while hypertension was the only pre-ECMO factor independently associated with in-hospital mortality (HR: 2.06, 95%CI: 1.06–4.00). High rates of bleeding (85.4%) and superinfections (91.7%) were recorded during ECMO, likely affecting the overall length of ECMO (18 days, IQR: 10–24) and the hospital stay (32 days, IQR: 24–47). Static lung compliance was lower in non-survivors (*p* = 0.031) and differed over time (*p* = 0.049), decreasing by 48% compared to initial values in non-survivors. **Conclusions:** Our data suggest the importance of considering NIS among the common ECMO eligibility criteria and changes in lung compliance during ECMO as a prognostic marker.

## 1. Introduction

Nowadays, the role of veno-venous extracorporeal membrane oxygenation (VV-ECMO) in managing acute respiratory distress syndrome (ARDS) is well established and supported by strong evidence [1]. In the context of COVID-19, ECMO was used in 7% of affected cases [2], and its efficacy remains debated due to the high variability in mortality rates—up to 90% in the earliest studies [3]—and the associated high costs, especially during the initial outbreak, when healthcare resources were strained [4].

However, patients with COVID-19-related ARDS (C-ARDS), which is refractory to conventional therapies (such as lung protective ventilation, neuromuscular blockade, high positive end-expiratory pressure (PEEP), lung recruitment maneuvers, prone positioning, and pulmonary vasodilators), faced high mortality rates ranging from 61% to 81% [5,6,7]. Consequently, the most important international organizations—including the Extracorporeal Life Support Organization (ELSO), World Health Organization (WHO), and Surviving Sepsis Campaign—recommended ECMO as a rescue therapy but only in selected cases and preferably in experienced centers [8,9,10].

In this study, we describe the clinical characteristics and 60-day clinical outcomes of a cohort of C-ARDS patients treated with VV-ECMO at our tertiary referral high-volume ECMO center. We focus on factors that may impact outcomes and describe the time course of lung mechanics parameters to assess disease evolution during extracorporeal support.

## 2. Materials and Methods

### 2.1. Study Design

The study was prospective, observational, and monocentric and performed in the university intensive care unit (ICU) at the “Città della Salute e della Scienza” Hospital in Turin (Italy) between March 2020 and December 2021, with the approval of the local ethic committee (protocol code 00103/2020).

All consecutive adult patients undergoing VV-ECMO for refractory hypoxemia due to severe C-ARDS were enrolled. The only exclusion criterion was denied informed consent.

### 2.2. Patients and ECMO Management

Since the influenza A(H1N1) pandemic in 2009, our ICU has been part of the national network of ECMO referral centers (ECMOnet), where all patients suffering from severe ARDS are transferred to receive specialized cares, including ECMO [11]. Suitability for the extracorporeal support is always assessed according to the updated Extracorporeal Life Support Organization (ELSO) guidelines [8]. Cannulation was performed at the patient’s bedside by a trained team composed of a cardiac surgeon, an intensivist, a perfusionist, and a nurse, all equipped with appropriate personal protective equipment (PPE) [12]. Drainage and perfusion cannulas were placed percutaneously through femoral veins using the ultrasound-guided Seldinger approach and serial dilations. In some cases, the perfusion cannula was inserted into the right internal jugular vein. Drainage cannulas had diameters of 21–25 Fr, while the perfusion ones had diameters of 19–23 Fr based on patients’ body size and vessels diameter at ultrasound examination. Unfractionated heparin was intravenously administered as a bolus of 50–100 U/kg before cannulas insertion and then titrated as a continuous infusion to achieve an activated clotting time (ACT) of 180–220 s, according to the ELSO protocol [13,14]. After cannulation, patients were connected to heparin-coated ECMO circuits powered by a Cardiohelp^®^ (Getinge—Göteborg, Sweden) centrifugal pump.

All patients received sedation and neuromuscular blockade to ensure their comfort and the best compliance with treatment. An ultra-protective strategy of mechanical ventilation (tidal volume ≤4 mL/kg of predicted body weight (PBW) to maintain plateau pressure ≤ 25 cmH_2_O and a respiratory rate of 8–10 breaths per minute) was applied to ensure lung rest, minimizing the risk of ventilator-induced lung injury (VILI) and biotrauma [15,16,17,18]. ECMO blood flow and gas flow were carefully regulated to achieve the desired respiratory goals. Weaning from ECMO was undertaken after the resolution of hypoxemia and a successful trial of stopping gas and oxygen delivery from the device for at least 24 h.

Tracheostomy was performed to facilitate ventilator discontinuation in more complex patients, guided by clinical judgement.

### 2.3. Outcomes and Data Collection

The primary outcome was mortality at 60 days after ECMO initiation, regardless of the cause of death. Secondary outcomes were ICU and hospital length of stay, rate of weaning from ECMO and mechanical ventilation at 60 days post ECMO, incidence of complications, lung mechanics and arterial blood gases parameters, predictors of mortality, and differences in clinical characteristics and outcomes between the first wave (defined from March to August 2020) and subsequent waves (defined from September 2020 to December 2021) of the COVID-19 outbreak.

Data were collected anonymously from patients’ clinical records and included information on age, sex, body mass index (BMI), comorbidities and habits such as active smoking before hospital admission, SOFA (sequential organ failure assessment) and APACHE II (acute physiologic assessment and chronic health evaluation II) scores at ICU admission, and pre-ECMO clinical conditions and management, including the presence of bacterial co-infections, type and mode of ventilatory supports, arterial blood gases values, targeted medications (steroids, Tocilizumab, hyperimmune plasma, and Remdesevir), and rescue therapies (lung recruitment maneuvers, pronation, and inhaled nitric oxide).

Timelines from the onset of COVID-19-related symptoms, hospitalization, and ICU-admission to ECMO implantation (day 0) and length of invasive mechanical ventilation (IMV) and ECMO were registered as well as ICU and hospital length of stay. After ECMO initiation, ventilator setting, lung mechanics, and arterial blood gases parameters were monitored from day 1 to day 14. Finally, ECMO-related adverse events (such as cannula thrombosis, membrane clotting, and circuit change) and clinical complications (such as bleeding, infections, pulmonary thromboembolism, deep venous thrombosis, and kidney failure requiring or not dialysis) were recorded.

### 2.4. Statistical Analysis

Continuous variables are expressed as median and interquartile range (IQR), and categorical variables are expressed as frequency and percentage. Comparison between two groups was performed for continuous and categorical variables with *t*-test or Wilcoxon–Mann–Whitney and chi-squared test or Fisher’s exact test, respectively.

Survival analysis was performed using Kaplan–Meier estimates during the period from ECMO initiation to 60 days later. In multivariable analysis, hazard ratios (HR) and 95% confidence intervals (95% CI) were estimated using the Cox regression model. The included pre-ECMO variables were defined based on the literature dealing with ECMO and COVID-19 patients and considering those variables with *p*-value < 0.20 in the univariate analysis (age, hypertension, ICU days before ECMO, use of steroids and Tocilizumab, NIS, and the Murray score) [19,20].

The occurrences of weaning from ECMO and IMV at 60 days after the beginning of extracorporeal support were assessed by calculating the cumulative incidence, considering death as a competitive event.

Data including ventilator setting, lung mechanics, and arterial blood gases after ECMO initiation were compared over time (from day 1 to day 14) and between survivor and non-survivor patients using analysis of variance (ANOVA) for repeated measures.

The level of statistical significance was set at 0.05, and tests were two-tailed. Analyses were performed using SPSS version 28.0 (Armonk, NY, USA: IBM Corp) and SAS statistical software version 9.4 (SAS Institute, Cary, NC, USA).

## 3. Results

### 3.1. Study Population

During the study period, 48 adult patients with C-ARDS received VV-ECMO support at our institution (Table 1). They were predominantly male (77.1%), with a median age of 54 (IQR: 49–61) years and a BMI of 29.6. In all, 85.4% of patients presented at least one comorbidity before extracorporeal support initiation: Obesity (defined as BMI ≥ 30) and hypertension had the highest incidence (more than 40%). Nineteen patients (39.6%) had a bacterial co-infection—defined by the presence of clinical and microbiological infection criteria at the time of ECMO cannulation or by its onset within 48 h of ECMO placement—without septic shock; *Staphylococcus aureus* was the most commonly cultured organism, and three patients were colonized by multi-drug-resistant (MDR) Gram-negative bacteria (Table S1). In 41 cases (85.4%), extracorporeal support was connected in peripheral hospitals, and then, the patients were centralized.

The times from onset of COVID-19-related symptoms and from ICU admission to the ECMO implantation day were longer for non-survivor patients (17 days, IQR: 11–22 vs. 15 days, IQR: 13–19 and 7 days, IQR: 4–11 vs. 4 days, IQR: 2–8, respectively), although there was no statistically significant difference (Table 1, Figure S1).

### 3.2. Supportive Care and Medications Delivered before ECMO Initiation

Most of the patients (89.6%) received non-invasive ventilatory support (NIS)—high-flow nasal cannula (HFNC), continuous positive airway pressure (CPAP), and non-invasive ventilation (NIV)—before orotracheal intubation, with different rates between survivor and non-survivor patients (57.1% vs. 95.1%, respectively, *p* = 0.018) (Table 1).

All patients underwent endotracheal intubation and neuromuscular blockade before ECMO. Median time from orotracheal intubation to ECMO initiation was 4 days (IQR: 2–7) in survivors and 5 days (IQR: 3–8) in non-survivors. The total time spent on ventilatory support (defined as NIS plus IMV days) before ECMO was longer in non-survivor patients (11 days, IQR: 8–15) than in survivors (8 days, IQR: 7–13) but without statistically significant difference (Table 1).

Lung recruitment maneuvers, pronation, and inhaled nitric oxide were used in 23 (48%), 43 (90%), and 17 (35%) patients, respectively, not reporting differences between survivors and non-survivors. Patients were treated with COVID-19-targeted medications such as steroids (32 patients, 66.7%), Tocilizumab (13 patients, 27.1%), and Remdesevir (14 patients, 29.2%). Only the use of steroids differed between survivor and non-survivor patients (28.6% vs. 73.2%, *p* = 0.033) (Table 1).

### 3.3. Pre-ECMO Ventilator Setting and Arterial Blood Gases

Pre-ECMO ventilator settings and arterial blood gases values are shown in Table 1. The median PaO_2_/FiO_2_ ratio was 64 mmHg (IQR: 55–72), and static lung compliance was 29.2 mL/cmH_2_O (IQR: 23.2–37.8), with no significant differences between survivor and non-survivor patients. Ventilator settings were protective, including a tidal volume of about 6 mL/kg of predicted body weight (PBW), plateau pressure of less than 30 cmH_2_O, and a median driving pressure of 16 cmH_2_O. The median Murray score for acute lung injury of all patients, assessed before ECMO initiation, was 3.3 (IQR: 3.0–3.6), and it was similar between survivors and non-survivors (Table 1).

### 3.4. Clinical Outcomes

**Figure 1:**
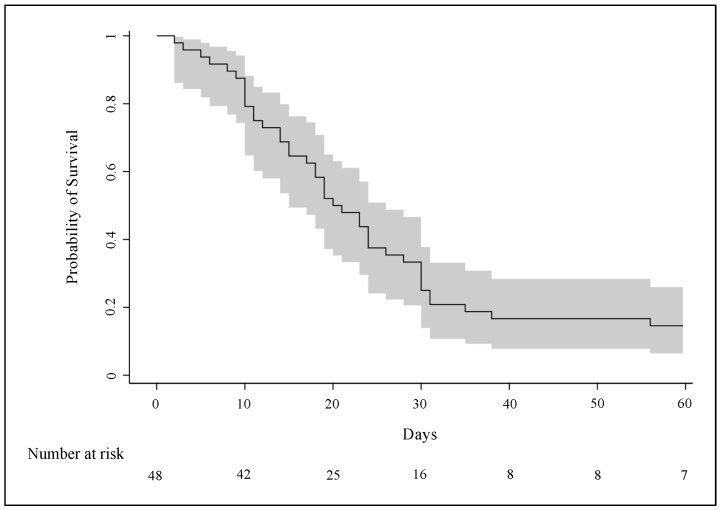
Kaplan–Meier survival curve of C-ARDS patients from the time of VV-ECMO implantation (day 0). The continuous line represents the probability of survival, while the grey area is the 95%CI. List of abbreviations: C-ARDS, COVID-19-related acute respiratory distress syndrome; VV-ECMO, veno-venous extracorporeal membrane oxygenation; CI, confidence interval.

**Figure 2:**
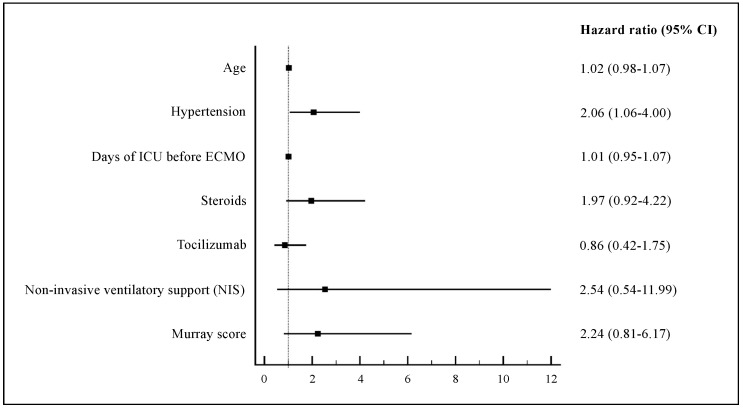
Cox model for factors associated with 60-day mortality after VV-ECMO implantation in patients affected by C-ARDS. All factors refer to the period before ECMO implantation. List of abbreviations: C-ARDS, COVID-19-related acute respiratory distress syndrome; VV-ECMO, veno-venous extracorporeal membrane oxygenation; ICU, intensive care unit; NIS, non-invasive ventilatory support.

According to the Kaplan–Meier curve, in-hospital mortality after 60 days from ECMO implantation was 85.4% (95% CI: 75.4–95.4) (Figure 1). The Cox model showed that hypertension was significantly associated with increased in-hospital mortality (HR: 2.06, 95% CI: 1.06–4.00), while days of ICU stay; use of NIS, steroids, and tocilizumab before ECMO initiation; and Murray score were not (Figure 2).

Median ICU and hospital length of stay were 26 (IQR: 19–39) and 32 days (IQR: 24 -47), respectively, in the overall population and 45 (IQR: 35–57) and 63 days (IQR: 54–76) in survivor patients.

**Figure 3:**
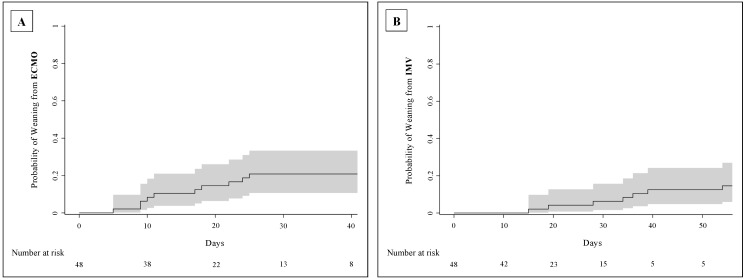
Cumulative incidence curves of weaning from ECMO (**A**) and invasive mechanical ventilation (**B**) of C-ARDS patients from the time of VV-ECMO implantation (day 0). The continuous lines represent the probability of weaning, considering death as a competitive event, while the grey areas are the 95%CI. List of abbreviations: C-ARDS, COVID-19-related acute respiratory distress syndrome; VV-ECMO, veno-venous extracorporeal membrane oxygenation; CI, confidence interval; IMV, invasive mechanical ventilation.

Median length of ECMO support was 18 days (IQR: 10–24) for all patients and 19 days (IQR: 9–24) for survivors, while IMV lasted a median of 25 days (IQR: 16–33) in all patients and 35 days (IQR: 25–42) in survivors. The estimated incidences of weaning from ECMO and from IMV 60 days after ECMO implantation were 23% (95% CI: 11.0–34.8) and 15% (95% CI: 4.6–24.6), respectively (cumulative incidence curves in Figure 3).

Tracheostomy was performed in 16 patients (33.3%)—of whom 6 survived—after a median time of 17 days (IQR: 11–21), following the initiation of IMV (Table 2).

### 3.5. Complications after ECMO Initiation

The incidence of complications did not differ significantly between survivor and non-survivor patients (Table 2). The main one was bleeding, which affected 85.4% of patients, with a higher rate in non-survivors (90.2% vs. 57.1%, *p* = 0.053). The most frequent bleeding sites were the airways (50%) and the insertion sites of ECMO cannulas (54%). Deep venous thrombosis occurred more frequently in survivors (42.9% vs. 4.9%, *p* = 0.018). Superinfections after ECMO implantation—such as ventilator-associated pneumonia (VAP) and bloodstream infection (BSI)—occurred in 91.7% of patients, with a higher rate in non-survivors (92.7% vs. 85.7%, *p* = 0.480) as well as for septic shock. Acute kidney failure was reported in 21 (43.8%) patients, requiring dialysis in 11 (22.9%).

### 3.6. Time Course of Respiratory Mechanics and Arterial Blood Gases Variables during ECMO Support

**Figure 4:**
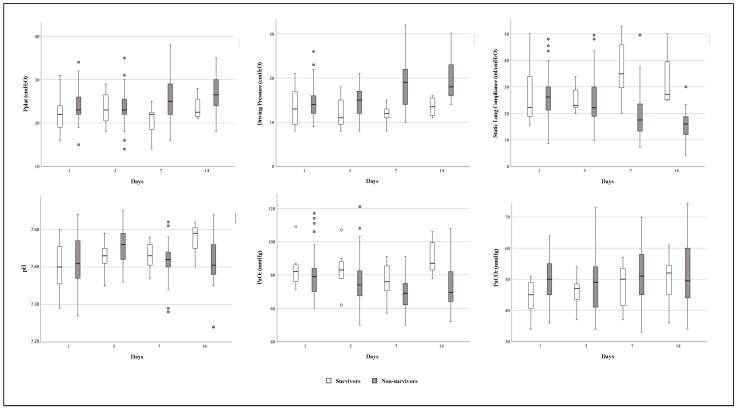
Trends of respiratory mechanics and arterial blood gases variables from day 1 (24 h after VV-ECMO implantation) to day 14 in survivor (white) and non-survivor (grey) C-ARDS patients. In the time-dependent analysis, static lung compliance differed significantly between survivors and non-survivors (*p* = 0.031) and over time (*p* = 0.049), driving pressure only over time (*p* = 0.030), and PaO_2_ only between survivors and non-survivors (*p* = 0.003). List of abbreviations: VV-ECMO, veno-venous extracorporeal membrane oxygenation; C-ARDS, COVID-19-related acute respiratory distress syndrome; P_plat_, plateau pressure; PaO_2_, arterial pressure of oxygen; PaCO_2_, arterial pressure of carbon dioxide.

In the time-dependent analysis, static lung compliance differed significantly between survivor and non-survivor patients (*p* = 0.031) and over time (*p* = 0.049). The compliance values of non-survivors decreased by 48% (IQR: 20–60%) from day 1 to day 14 after ECMO cannulation; the driving pressure values increased over time (*p* = 0.030), especially in non-survivor patients, with a rate of 43% (IQR: 0–78%) from day 1 to day 14, while the PaO_2_ values differed between survivors and non-survivors (*p* = 0.003), with a constant trend over time in the two groups (*p* = 0.097) (Figure 4).

The time course of ventilator and ECMO setting parameters did not show significant differences, except for the respiratory rate and ECMO sweep gas, which increased significantly over time to adjust PaCO_2_ values. PEEP values decreased over time, especially in non survivors (Table S2).

### 3.7. Differences between Patients Treated in the First and Subsequent Waves of COVID-19 Outbreak

In our study population, 14 (29.2%) patients were enrolled during the first wave of the COVID-19 outbreak and 34 (70.8%) in the subsequent ones. The comparison between patients treated in the two different periods highlighted that, during the first wave, extracorporeal support was more frequently applied to subjects without any comorbidities and with a lower incidence of bacterial co-infections (35.7% vs. 5.9%, *p* = 0.017, and 21.4% vs. 47.1%, *p* = 0.099, respectively) (Table S3). On the contrary, the use of NIS before endotracheal intubation was reported more frequently after the first wave (71.4% vs. 97.1%, *p* = 0.021). Additionally, IMV was shorter (3 days, IQR: 2–6 vs. 7 days, IQR: 4–9, *p* = 0.021), although pre-ECMO total ventilation days (NIS plus IMV) did not differ between the two periods. Similarly, during the subsequent waves, a more protective ventilatory strategy was applied to patients before ECMO implantation (lower median tidal volume, 6.6 vs. 7.2 mL/kg, *p* = 0.038, and plateau pressure, 27 vs. 29 cmH_2_O, *p* = 0.027). Lung recruitment maneuvers were used more often in the first wave (85.7% vs. 32.4%, *p* = 0.001), while steroids were administered more frequently during the subsequent waves (79.4% vs. 35.7%, *p* = 0.004).

ICU and hospital length of stay were longer during the first wave (median 38 vs. 24 days and 44 vs. 28 days, respectively, *p* = 0.005) as well as the length of ECMO and IMV (median 22 vs. 14 days, *p* = 0.012, and 35 vs. 23 days, *p* = 0.002, respectively), while 60-day mortality after ECMO cannulation was higher in patients enrolled during the subsequent waves (88.2% vs. 78.6%) (Table S4).

Rates of clinical complications were similar between the first and subsequent waves, except for superinfections due to MDR bacteria, which occurred more frequently during the subsequent waves (73.5% vs. 35.7%, *p* = 0.014), when patients in our ICU were more frequently colonized by MDR Gram-negative bacteria (88.2% vs. 35.7%, *p* = 0.001), mainly by carbapenem-resistant *Acinetobacter baumannii* (59% of cases) (Table S4).

## 4. Discussion

### 4.1. In-Hospital Mortality and Pre-ECMO Clinical Characteristics

The present study, designed to evaluate the outcomes of a cohort of C-ARDS patients undergoing VV-ECMO during the COVID-19 outbreak, evidenced a particularly high mortality at 60 days after ECMO initiation (85.4%, 95% CI: 75.4–95.4). These data align with the earliest studies performed in C-ARDS patients treated with ECMO [3]. Subsequent studies reported more favorable outcomes, with mortality rates between 30–55% [21,22,23,24,25,26], closer to those described for non-C-ARDS patients undergoing ECMO before the outbreak (40% in the ELSO registry) [8]. However, it should be noted that non-survivor patients treated with VV-ECMO for severe C-ARDS were typically characterized by greater complexity due to older age, multiple comorbidities, and a high need for vasopressors and dialysis [27].

Our cohort of patients was certainly characterized by severe baseline conditions before ECMO connection, as evidenced by high median SOFA and APACHE II scores of 10 and 24 points, respectively, which are known to be associated with a predicted ICU mortality greater than 50% even without ECMO. The non-survivor subgroup was older—although the difference did not reach statistical significance—and this finding is in agreement with the ELSO registry, where increasing age was associated with a higher in-hospital mortality risk [21]. Additionally, 39.6% of our patients had pre-existing bacterial co-infections, in line with the literature (about 37% [21]), indicating that co-infection did not preclude ECMO, despite its higher incidence in non-survivors (41.5% vs. 28.6%).

Among pre-ECMO characteristics, only the presence of hypertension (43.8% of the patients) was associated with increased in-hospital mortality (HR: 2.06, 95% CI: 1.06–4.00), consistent with other reports suggesting that hypertension could be associated with a worse prognosis in COVID-19. Uncontrolled systolic blood pressure has been shown to worsen COVID-19 progression by causing vascular remodeling and endothelial damage, exacerbated by the inflammatory state promoted by the ECMO circuit [28].

The severity of our study population was also confirmed by a lower median PaO_2_/FiO_2_ ratio before ECMO implantation (64 mmHg, IQR: 55–72) compared to the one observed in the ELSO population (72 mmHg, IQR: 70–93) [21], although it was comparable to the ratio reported in a cohort of Italian COVID-19 patients undergoing ECMO (62 mmHg, IQR: 56–80) [29]. Other pre-ECMO gas exchange and lung mechanics parameters were consistent with data described in the literature and did not differ between survivors and non-survivors [15,30], although recent observational studies reported that in critically ill COVID-19 patients, lower pH and higher tidal volume and plateau pressure were associated with unfavorable outcomes [31,32].

Another contributing factor to the baseline severity of our population was the high number of patients (85.4%) who required centralization to our ICU due to refractory respiratory failure, resulting in a longer time before undergoing ECMO support. This factor aligns with data described by Lebreton and colleagues, who reported a 90-day mortality of 54% with shorter median times from COVID-19 onset and hospitalization to ECMO cannulation than ours (14 vs. 17 and 7 vs. 12 days, respectively) [23].

### 4.2. Supportive Cares and Medications before ECMO Implantation

Factors related to supportive care administered before ECMO could affect in-hospital mortality. Our study population received non-invasive ventilatory support—particularly CPAP—more frequently than the cohort of C-ARDS patients described in the ELSO registry (89.6% vs. 56%) [21]. Moreover, non-survivor patients underwent NIS more often than survivors (95.1% vs. 57.1%, *p* = 0.018) and experienced more total ventilation (NIS plus IMV) days (median of 11 vs. 8 days), although multivariate analysis excluded an independent role of NIS on mortality. The real impact of NIS before ECMO on clinical outcomes has not been clearly defined. However, it is known that prolonged NIS could worsen lung injury due to “self-induced lung injury” (SILI) [33]. Boscolo et al. reported that NIV support longer than 48 h in C-ARDS patients before ICU admission was independently associated with increased in-hospital mortality [34]. Ahmad et al. demonstrated that patients undergoing three or more days of NIS before intubation and ECMO showed worse lung damage, prolonged weaning from ECMO and mechanical ventilation, and longer hospital stay [35]. These data seem to suggest a role of NIS in affecting the outcomes of C-ARDS patients undergoing ECMO and support the hypothesis of considering the time spent on non-invasive ventilation in the assessment of ECMO suitability.

Among COVID-19-targeted therapies, steroids were the most commonly administered to our population (66.7%), with a higher rate in non-survivors (73.2% vs. 28.6%, *p* = 0.033). However, steroid use was not associated with mortality in the Cox model. During the COVID-19 outbreak, dexamethasone was found to reduce mortality in those patients requiring supplemental oxygen by mitigating the inflammatory response secondary to viral infection [36]. The high mortality observed in patients treated with steroids may be explained by their more compromised conditions at ECMO initiation; moreover, immunomodulatory therapies could significantly impact lymphocytopenia, reduce viral clearance, and increase the risk of nosocomial infections, resulting in negative outcomes [37].

### 4.3. Complications after ECMO Initiation

Complications occurring after ECMO initiation may have also affected patient outcomes. Critically ill COVID-19 patients, particularly those treated with ECMO, experienced high incidences of complications such as bleeding, thromboembolism, and superinfections due to prolonged intubation and hospital stay, use of large-caliber circuit catheters, continuous anticoagulant therapies, frequent bacterial colonization or co-infections before ECMO, and the extensive use of immunotherapies including steroids [27]. However, the actual rate of complications in patients treated with VV-ECMO was similar between COVID-19 and non-COVID-19 populations [19].

We observed higher incidences of bleeding and superinfections (85.4% and 91.7%, respectively), and although rates did not differ between survivors and non-survivors, these complications likely contributed to the increased observed mortality. In C-ARDS patients undergoing ECMO, bleeding and superinfections (both ventilatory-acquired pneumonia and bloodstream infection) have already been associated with longer ECMO duration, longer ICU and hospital stay, and higher in-hospital mortality [38,39].

### 4.4. Length of ECMO Support and Other Clinical Outcomes

The length of ECMO and invasive mechanical ventilation as well as ICU and hospital length of stay were longer in our studied population than those observed in non-COVID-19 patients and in other COVID-19 experiences [19,21,29]. In previous outbreaks, the ECMO duration was reported to be 13 days for H1N1 influenza in Italy [11], 10 days for MERS [40], and 15 and 9 days for ARDS patients in the EOLIA and CESAR trials, respectively [15,41]. According to the ELSO registry, C-ARDS patients spent a median of 14 days on VV-ECMO [21]. In our population, 23% of patients were discontinued from ECMO, and survivors spent a median of 19 days (IQR: 9–24) on ECMO, resulting in more days of invasive mechanical ventilation and hospitalization. Specifically, the median length of hospital stay was 32 days (IQR: 24–47) in our study compared to 27 days in the ELSO registry. These differences may be attributed to a greater severity of SARS-CoV-2 infection, resulting in worse lung impairment and complications, and to the high baseline severity observed in our population.

### 4.5. Evolution of Respiratory Mechanics during ECMO Support

ECMO support allowed us to apply an ultra-protective ventilatory strategy to C-ARDS patients, decreasing plateau pressure and reducing the risk of barotrauma and biotrauma, in line with the management of VV-ECMO patients before the COVID-19 outbreak [16]. However, respiratory mechanics worsened from day 1 to day 14 after ECMO initiation, especially in the non-survivor cohort, where static lung compliance decreased by 48%, and driving pressure increased by 43% compared to baseline values. This finding could be associated with worse lung damage due to the specific severity of COVID-19 [40]. Moreover, static lung compliance differed between survivor and non-survivor patients after seven days of treatment, decreasing severely in non-survivors and potentially indicating a therapeutic failure.

### 4.6. Patient Management in First Versus Subsequent Waves of COVID-19 Outbreak

Over the course of the COVID-19 outbreak, the use of VV-ECMO for severe C-ARDS patients increased, primarily due to a rise in the number of centers providing extracorporeal support. This led to changes in C-ARDS patient selection, treatments, and outcomes between the first and subsequent waves [26,42]. After the first wave, more patients with major comorbidities, high clinical assessment scores, and pre-existing bacterial co-infections were selected for ECMO. Additionally, there was an expansion in the use of non-invasive ventilatory supports (especially HFNC and CPAP) before tracheal intubation and a reduced length of invasive mechanical ventilation, suggesting an earlier utilization of ECMO. A more protective ventilatory strategy was also applied before ECMO, with limited use of lung recruitment maneuvers, considering the possibility of developing a C-ARDS pattern characterized by low lung “recruitability” [43].

The use of ECMO in patients with lower survival chances, greater and longer use of non-invasive ventilatory supports, and consequent increased risk of SILI, along with higher rates of superinfections (especially by MDR pathogens) and septic shock, were all factors that possibly contributed to the increased mortality rate of patients enrolled after the first pandemic wave (88.2% vs. 78.6%), consistent with the literature [26,42].

Furthermore, although in the current study, we were unable to identify the specific SARS-CoV-2 variants affecting the patients, we speculate that higher mortality rate might be a consequence of a greater spread of the delta variant in Europe after the first pandemic wave [26]. The delta variant was more virulent and caused an overwhelming inflammatory response that might explain the excess mortality of delta-infected patients compared to those affected by the wild-type SARS-CoV-2 strain [26].

### 4.7. Limitations

Our study has some limitations. First, we described a single-center population. We are a tertiary referral, high-volume ECMO center in the north of Italy, and our findings are not replicable in other smaller centers and regions with different resources. Second, the analyzed sample size was limited, and the small number of enrolled cases represents an important limitation of the study. Therefore, the power of the study may not be sufficient to identify mortality-associated risk factors. However, we evaluated and recorded extensive data on patients’ baseline severity and pre-hospital clinical characteristics, providing an accurate description of the studied population. Third, data collection was carried out from the beginning of the outbreak to December 2021; thus, final outcomes might be affected by changes in patient management due to the numerous clinical trials published during the pandemic and the varying availability of new targeted treatments, particularly antivirals and immunotherapies. This limitation could be mitigated by comparing data between the first and subsequent waves, showing that, despite the expansion of COVID-19-targeted therapies such as Remdesivir and dexamethasone, the mortality rate of our patients under VV-ECMO remained high even after the first wave, consistent with other international studies [42]. Fourth, clinical outcomes were evaluated only up to 60 days after ECMO initiation, and we did not investigate patients’ post-hospitalization outcomes. Recent studies have reported that proper patient selection and management of extracorporeal support in severe C-ARDS patients can lead to high survival rates even one year after ECMO cannulation [44]. Finally, as the study was not a randomized clinical trial, it was not possible to draw definitive conclusions on the benefits of VV-ECMO in patients with C-ARDS.

## 5. Conclusions

The present study revealed a particularly high 60-day mortality rate (85.4%) in C-ARDS patients treated with VV-ECMO during the COVID-19 outbreak. This elevated mortality could be attributed to patients’ more severe baseline conditions and higher rates of complications, including bleeding and infections, which may also impact ECMO and IMV duration as well as ICU and hospital length of stay. Hypertension emerged as the only pre-ECMO factor independently associated with increased mortality. Non-survivor patients were more frequently treated with non-invasive ventilatory support and steroids. Lung system compliance significantly differed between survivors and non-survivors, particularly after seven days of treatment, with a 48% decrease compared to initial values.

Further investigations are necessary to confirm whether VV-ECMO offers better outcomes than conventional therapies for severe C-ARDS patients, the role of non-invasive ventilatory support within the ECMO eligibility criteria, and the potential role of lung compliance reduction after ECMO initiation in defining a therapeutic failure.

## Figures and Tables

**Table 1:** Baseline characteristics of patients affected by C-ARDS before VV-ECMO support.

Variables	Total (n = 48)	Survivors (n = 7)	Non-Survivors (n = 41)	*p*-Value
**Age**, ***years***, *median (IQR)*	54 (49–61)	50 (48–53)	55 (50–61)	0.457
**Sex**, ***male***, *n* (%)	37 (77.1)	6 (85.7)	31 (75.6)	0.557
**BMI**, **kg/m^2^**, *median (IQR)*	29.6 (27.5–33.9)	31.8 (28.4–34.7)	29.4 (27.1–33.3)	0.430
**Pre-existing comorbidities:**				
**No comorbidity**, *n* (%)	7 (14.6)	2 (28.6)	5 (12.2)	0.267
**Obesity**, *n* (%)	23 (47.9)	4 (57.1)	19 (46.3)	0.696
**Active smoking**, *n* (%)	8 (16.7)	1 (14.3)	7 (17.1)	0.855
**Hypertension**, *n* (%)	21 (43.8)	1 (14.3)	20 (48.8)	0.089
**CAD**, *n* (%)	1 (2.1)	0	1 (2.4)	1.000
**Lung disease (asthma/COPD)**, *n* (%)	6 (12.5)	0	6 (14.6)	0.279
**Diabetes mellitus**, *n* (%)	7 (14.6)	0	7 (17.1)	0.237
**Hypothyroidism**, *n* (%)	4 (8.3)	0	4 (9.8)	1.000
**Chronic immunosuppression**, *n* (%)	1 (2.1)	0	1 (2.4)	1.000
**Severity score at ECMO implantation:**				
**SOFA**	10 (8–12)	12 (7–12)	10 (8–12)	0.309
**APACHE II**	24 (19–26)	25 (20–26)	24 (19–25)	0.499
**Pre-existing bacterial co-infection**, *n* (%)	19 (39.6)	2 (28.6)	17 (41.5)	0.687
**Days from COVID-19 onset to ECMO**, *median (IQR)*	17 (12–22)	15 (13–19)	17 (11–22)	0.835
**Days from hospitalization to ECMO**, *median (IQR)*	12 (7–17)	11 (6–12)	12 (8–17)	0.412
**Days of ICU before ECMO**, *median (IQR)*	7 (3–11)	4 (2–8)	7 (4–11)	0.151
**Mobile ECMO**, *n* (%)	41 (85.4)	7 (100)	34 (82.9)	0.237
**Non-invasive ventilatory support (NIS) before intubation**, *n* (%):	43 (89.6)	4 (57.1)	39 (95.1)	0.018
**HFNC**, *n* (%)	6 (12.5)	0	6 (14.6)	
**CPAP**, *n* (%)	34 (70.8)	3 (42.9)	31 (75.6)	
**NIV**, *n* (%)	15 (31.3)	2 (28.6)	13(31.7)	
**Days of NIS**, *median (IQR)*	5 (3–10)	6 (0–10)	5 (3–9)	0.519
**Days of invasive mechanical ventilation (IMV)**, *median (IQR)*	5 (3–8)	4 (2–7)	5 (3–8)	0.527
**Total days of ventilation (NIS + IMV)**, *median (IQR)*	11 (8–15)	8 (7–13)	11 (8–15)	0.355
**Rescue therapies:**				
**LRM**, *n* (%)	23 (47.9)	3 (42.9)	20 (48.8)	1.000
**Pronation**, *n* (%)	43 (89.6)	6 (85.7)	37 (90.2)	0.562
**iNO**, *n* (%)	17 (35.4)	3 (42.9)	14 (34.1)	0.686
**COVID-19-targeted therapies:**				
**Steroids**, *n* (%)	32 (66.7)	2 (28.6)	30 (73.2)	0.033
**Tocilizumab**, *n* (%)	13 (27.1)	0	13 (31.7)	0.081
**Hyperimmune plasma**, *n* (%)	6 (12.5)	1 (14.3)	5 (12.2)	1.000
**Remdesevir**, *n* (%)	14 (29.2)	2 (28.6)	12 (29.3)	1.000
**Pre-ECMO ventilator setting and arterial blood gases:**				
**TV/PBW**, **mL/kg**, *median (IQR)*	6.7 (6.1–7.4)	6.3 (6.0–7.0)	6.7 (6.1–7.5)	0.143
**RR**, ***breaths/min***, *median (IQR)*	28 (24–30)	30 (28–35)	26 (24–30)	0.286
**PEEP**, **cmH_2_O**, *median (IQR)*	12 (10–14)	10 (8–12)	12 (10–14)	0.311
**FiO_2_**, ***%***, *median (IQR)*	100 (100–100)	100 (85–100)	100 (100–100)	0.506
**P_plat_**, **cmH_2_O**, *median (IQR)*	28 (23–30)	29 (25–31)	28 (23–30)	0.700
**DP**, **cmH_2_O**, *median (IQR)*	16 (12–19)	17 (12–22)	16 (12–19)	0.820
**Static lung compliance**, **mL/cmH_2_O**, *median (IQR)*	29.2 (23.2–37.8)	28.5 (19–40.3)	29.2 (23.2–37.8)	0.703
**pH**, *median (IQR)*	7.34 (7.30–7.40)	7.33 (7.26–7.37)	7.34 (7.31–7.41)	0.433
**PaO_2_/FiO_2_**, **mmHg**, *median (IQR)*	64 (55–72)	64 (57–71)	64 (53–72)	0.941
**PaCO_2_**, **mmHg**, *median (IQR)*	59 (54–68)	55.4 (54–61)	59 (54–68)	0.353
**HCO^−^_3_**, **mmol**/L, *median (IQR)*	33 (29–36)	31 (26–36)	33 (30–36)	0.517
**Murray score**, *median (IQR)*	3.3 (3.0–3.6)	3.3 (3.0–3.5)	3.3 (3.3–3.7)	0.198

List of abbreviations: C-ARDS, COVID-19-related acute respiratory distress syndrome; VV-ECMO, veno-venous extracorporeal membrane oxygenation; IQR, interquartile range; BMI, body mass index; CAD, coronary artery disease; COPD, chronic obstructive pulmonary disease; SOFA, sequential organ failure assessment score; APACHE II, acute physiologic assessment and chronic health evaluation score II; ICU, intensive care unit; NIS, non-invasive ventilatory support; HFNC, high-flow nasal cannula; CPAP, continuous positive airway pressure; NIV, non-invasive ventilation; IMV, invasive mechanical ventilation; LRM, lung recruitment maneuvers; iNO, inhaled nitric oxide; TV/PBW, tidal volume divided by predicted body weight; RR, respiratory rate; PEEP, positive end-expiratory pressure; FiO_2_, inspired fraction of oxygen; P_plat_, plateau pressure; DP, driving pressure; PaO_2_, arterial pressure of oxygen; PaCO_2_, arterial pressure of carbon dioxide; HCO^−^_3_, plasma bicarbonate concentrations.

**Table 2:** Complications in C-ARDS patients treated with VV-ECMO.

Complications	Total (n = 48)	Survivors (n = 7)	Non-Survivors (n = 41)	*p*-Value
**ECMO-related:**				
**Cannula thrombosis**, *n* (%)	2 (4.2)	1 (14.3)	1 (2.4)	0.273
**Membrane clotting**, *n* (%)	14 (29.2)	1 (14.3)	13 (31.7)	0.349
**Circuit change**, *n* (%)	13 (27.1)	1 (14.3)	12 (29.3)	0.410
**Bleeding**, *n* (%)	41 (85.4)	4 (57.1)	37 (90.2)	0.053
**Hemorrhagic stroke**, *n* (%)	3 (6.3)	0	3 (7.3)	1.000
**Hemorrhagic shock**, *n* (%)	2 (4.2)	0	2 (4.9)	1.000
**Superinfection**, *n* (%)	44 (91.7)	6 (85.7)	38 (92.7)	0.480
**VAP**, *n* (%)	39 (81.3)	5 (71.4)	34 (82.9)	0.601
**BSI**, *n* (%)	18 (37.5)	4 (57.1)	14 (34.1)	0.400
**Septic shock**, *n* (%)	27 (56.3)	3 (42.9)	24 (58.5)	0.683
**Deep venous thrombosis**, *n* (%)	5 (10.4)	3 (42.9)	2 (4.9)	0.018
**Pulmonary thromboembolism**, *n* (%)	3 (6.3)	0	3 (7.3)	1.000
**Acute kidney disease**, *n* (%)	21 (43.8)	3 (42.9)	18 (43.9)	1.000
**Renal replacement therapy**, *n* (%)	11 (22.9)	2 (28.6)	9 (22.0)	0.653
**Tracheostomy**, *n* (%)	16 (33.3)	6 (85.7)	10 (24.4)	0.001

List of abbreviations: C-ARDS, COVID-19-related acute respiratory distress syndrome; VV-ECMO, veno-venous extracorporeal membrane oxygenation; VAP, ventilator-associated pneumoniae; BSI, bloodstream infection.

## Data Availability

The database generated during the study is available on appropriate request by writing to the corresponding author.

## Supplementary Materials

The following supporting information can be downloaded at: https://www.mdpi.com/article/10.3390/jcm13123545/s1: Table S1: Bacteria responsible for co-infection at the time of VV-ECMO initiation in C-ARDS patients; Table S2: Trends of ventilator and VV-ECMO setting parameters from day 1 (24 h after ECMO implantation) to day 14 in C-ARDS patients; Table S3: Comparison of the baseline characteristics of patients affected by C-ARDS before VV-ECMO implantation between the first and subsequent waves of COVID-19 outbreak; Table S4: Comparison of outcomes of C-ARDS patients treated with VV-ECMO between the first and subsequent waves of COVID-19 outbreak; Figure S1: Timeline from the start of COVID-19-related symptoms to VV-ECMO implantation in survivor (continuous arrow) and non-survivor (dashed arrow) C-ARDS patients.
