# Supplementary material for: Outcomes and Impact of Pre-ECMO Clinical Course in Severe COVID-19-Related ARDS Treated with VV-ECMO: Data from an Italian Referral ECMO Center
Source: J Clin Med. 2024 Jun 17;13(12):3545. doi: 10.3390/jcm13123545 (PMC11204940; doi:10.3390/jcm13123545)

SUPPLEMENTARY MATERIALS

Table S1. Bacteria responsible for co-infection at VV-ECMO initiation in C-ARDS patients.

	GRAM +	N	GRAM -	N	GRAM +/GRAM -	N
**PNEUMONIA**	*Streptococcus p.*	1	*Pseudomonas a.*	1		
	MSSA	4	*Serratia m.*	2		
	MRSA	2	*CR-Acinetobacter b.*	3		
			Klebsiella p.	1		
			CR-Kp	1		
**BSI**	*E. faecalis*	2				
**PNEUMONIA + BSI**			*CR-Kp*	1	*MRSA*<br>*CR-Acinetobacter b.*	1
**ENTERIC COLONIZATION***			*CR-Kp*	2		
			CR-Acinetobacter b.	1		

*Defined by pathogen isolation in rectal swabs.
List of abbreviations: VV-ECMO: veno-venous extracorporeal membrane oxygenation; C-ARDS: COVID-19 related acute respiratory distress syndrome; MSSA: *methicillin-susceptible Staphylococcus aureus;* MRSA*: methicillin-resistant Staphylococcus aureus*; CR-Acinetobacter b.: carbapenem-resistant Acinetobacter baumannii; CR-Kp: carbapenem-resistant Klebsiella pneumoniae; BSI: bloodstream infection.

Table S2. Trends of ventilator and VV-ECMO setting parameters from day 1 (24 hours after ECMO implantation) to day 14 in C-ARDS patients.

		Day 1 (N=48)	Day 3 (N=46; *deaths=2*)	Day 7 (N=44; *deaths=4*)	Day 14 (N=33; *deaths=15*)
**Ventilator setting parameters**					
**TV/PBW, *ml/Kg***	**Survivors**	4.2 (4 - 5.7)	5 (3.0 - 5.2)	5.9 (5.1 - 6.2)	5.7 (4.2 - 6.3)
	**Non-survivors**	5.4 (4.6 - 6.0)	5.1 (4.2 - 6.0)	4.7 (4.1 - 5.5)	4 (3.6 - 5.4)
**RR, *breaths/min* ***	**Survivors**	10 (8 - 10)	8 (8 - 10)	18 (8 - 22)	20 (14 - 26)
	**Non-survivors**	10 (8 - 10)	10 (8 - 10)	10 (10 - 15)	14 (10 - 16)
**FiO_{2VENT}, %**	**Survivors**	80 (60 - 100)	60 (50 - 90)	50 (45 - 60)	50 (30 - 70)
	**Non-survivors**	60 (50 - 75)	50 (50 - 80)	60 (50 - 80)	60 (50 - 90)
**PEEP, *cmH_2O* ***	**Survivors**	8 (8 - 10)	10 (10 - 15)	8 (6 - 10)	10 (8 - 10)
	**Non-survivors**	10 (8 - 12)	8 (6 - 12)	8 (5 - 10)	6 (5 - 10)
**ECMO setting parameters**					
**BF, *L/min***	**Survivors**	3.8 (3.8 - 4.8)	4.0 (4.0 - 4.3)	3.9 (3.0 - 4.3)	3.8 (3.6 - 5.5)
	**Non-survivors**	4.0 (3.5 - 4.3)	4.1 (3.6 - 4.5)	4.0 (3.3 - 4.6)	4.1 (3.5 - 4.9)
**SG, *L/min* ***	**Survivors**	4.0 (3.5 - 5.0)	5.0 (3.5 - 5.5)	5.2 (4.0 - 7.0)	6.5 (5.5 - 8.6)
	**Non-survivors**	3.5 (2.7 - 4.0)	4.0 (3.5 - 5.0)	5 (3.7 - 6.2)	6.2 (5.0 - 7.5)
**FiO_{2ECMO}, %**	**Survivors**	100 (80 - 100)	90 (50 - 100)	60 (45 - 100)	100 (75 - 100)
	**Non-survivors**	90 (80 - 100)	80 (70 - 100)	90 (75 - 100)	100 (70 - 100)

Variables are expressed as median (IQR).
* The parameter significantly differed over the time *($p<0.05$)*
List of abbreviations: C-ARDS: COVID-19 related acute respiratory distress syndrome; VV-ECMO: veno-venous extracorporeal membrane oxygenation; TV/PBW: tidal volume divided by predicted body weight; RR: respiratory rate; FiO_{2VENT}: ventilator inspired fraction of oxygen; PEEP: positive end-expiratory pressure; BF: blood flow; SG: sweep gas; FiO_{2ECMO}: ECMO inspired fraction of oxygen.

Table S3. Comparison of the baseline characteristics of patients affected by C-ARDS before VV-ECMO implantation between the first and subsequent waves of COVID-19 outbreak.

VARIABLES	FIRST WAVE* (N = 14)	SUBSEQUENT WAVES* (N = 34)	*p-value*
**Age,** ***years,*** *median (IQR)*	53 (50 - 55)	56 (49 - 61)	*0.601*
**Sex,** ***male,*** *n (%)*	11 (78.6)	26 (76.5)	*0.875*
**BMI,** ***Kg/m²,*** *median (IQR)*	28.4 (27.6 - 33.5)	30.9 (26.6 - 34.3)	*0.812*
**Pre-existing comorbidities:**			
**No comorbidity,** *n (%)*	5 (35.7)	2 (5.9)	*0.017*
**Obesity,** *n (%)*	5 (35.7)	18 (52.9)	*0.278*
**Active smoking,** *n (%)*	2 (14.3)	6 (17.6)	*0.776*
**Hypertension,** *n (%)*	4 (28.6)	17 (50)	*0.174*
**CAD,** *n (%)*	0	1 (2.9)	*1.000*
**Lung disease (asthma/COPD),** *n (%)*	1 (7.1)	5 (14.7)	*0.656*
**Diabetes Mellitus,** *n (%)*	2 (14.3)	5 (14.7)	*1.000*
**Hypothyroidism,** *n (%)*	2 (14.3)	2 (5.9)	*0.569*
**Chronic immunosuppression,** *n (%)*	0	1 (2.9)	*1.000*
**Severity score:**			
**SOFA**	10 (7 - 12)	10 (8 - 12)	*0.680*
**APACHE II**	24 (22 - 25)	24 (19 - 25)	*0.632*
**Pre-existing bacterial co-infection,** *n (%)*	3 (21.4)	16 (47.1)	*0.099*
**Days from COVID-19 onset to ECMO,** *median (IQR)*	17 (11 - 23)	17 (13 - 21)	*0.502*
**Days from hospitalization to ECMO,** *median (IQR)*	12 (6 - 17)	12 (8 - 17)	*0.982*
**Days of ICU before ECMO,** *median (IQR)*	8 (5 - 14)	6 (3 - 10)	*0.116*
**Mobile ECMO,** *n (%)*	10 (71.4)	31 (91.2)	*0.171*
**Non-invasive ventilatory support (NIS) before intubation,** *n (%)*:	10 (71.4)	33 (97.1)	*0.021*
**HFCN,** *n (%)*	1 (7.1)	5 (14.7)	
**CPAP,** *n (%)*	6 (42.9)	28 (82.4)	
**NIV,** *n (%)*	4 (28.6)	11 (32.4)	
**Days of NIS,** *median (IQR)*	3 (0 - 9)	6 (4 - 10)	*0.094*
**Days of invasive mechanical ventilation (IMV),** *median (IQR)*	7 (4 - 9)	3 (2 - 6)	*0.021*
**Total days of ventilation (NIS+IMV),** *median (IQR)*	11 (7 - 17)	11 (8 - 13)	*0.955*
**Rescue therapies:**			
**LRM,** *n (%)*	12 (85.7)	11 (32.4)	*0.001*
**Pronation,** *n (%)*	12 (85.7)	31 (91.2)	*0.621*
**iNO,** *n (%)*	4 (28.6)	13 (38.2)	*0.525*
**COVID-19 targeted therapies:**			
**Steroids,** *n (%)*	5 (35.7)	27 (79.4)	*0.004*
**Tocilizumab,** *n (%)*	6 (42.9)	7 (20.6)	*0.115*
**Hyperimmune plasma,** *n (%)*	0	6 (17.6)	*0.093*
**Remdesevir,** *n (%)*	6 (42.9)	8 (23.5)	*0.181*
**Pre-ECMO ventilator setting and arterial blood gases:**			
**TV/PBW,** ***ml/Kg,*** *median (IQR)*	7.2 (6.4 - 8.1)	6.6 (6.1 - 7)	*0.038*
**RR,** ***breaths/min,*** *median (IQR)*	25 (18 - 30)	28 (25 - 31)	*0.104*
**PEEP,** ***cmH_2O,*** *median (IQR)*	12 (10 - 15)	12 (8 - 12)	*0.103*
**FiO_2, %,** *median (IQR)*	100 (95 - 100)	100 (100 - 100)	*0.868*
**P_{plat},** ***cmH_2O,*** *median (IQR)*	29 (25 - 35)	27 (22 - 30)	*0.027*
**DP,** ***cmH_2O,*** *median (IQR)*	14 (13 - 22)	16 (12 - 19)	*0.256*
**Static lung compliance,** ***ml/ cmH_2O,*** *median (IQR)*	28.6 (20.5 - 40.4)	29.2 (24.6 - 37.1)	*0.671*
**pH,** *median (IQR)*	7.33 (7.28 - 7.38)	7.36 (7.30 - 7.41)	*0.199*
**PaO_2/FiO_2,** ***mmHg,*** *median (IQR)*	64 (55 - 70)	64 (54 - 72)	*0.904*
**$PaCO_2$,** ***mmHg,*** *median (IQR)*	59 (53 - 73)	59 (54 - 68)	*0.958*
**HCO^-_3,** ***mmol/L,*** *median (IQR)*	31 (27 - 35)	33 (31 - 37)	*0.192*
**Murray score,** *median (IQR)*	3.4 (3.3 - 3.8)	3.3 (3.0 - 3.5)	*0.157*

* 'First wave' was defined as the pandemic time between March and August 2020; 'Subsequent waves' comprehended the time between September 2020 and December 2021.
List of abbreviations: C-ARDS: COVID-19 related acute respiratory distress syndrome; VV-ECMO: veno-venous extracorporeal membrane oxygenation; IQR: interquartile range; BMI: body mass index; CAD: coronary artery disease; COPD: *chronic obstructive pulmonary disease; SOFA:* sequential organ failure assessment score; APACHE II: acute physiologic assessment and chronic health evaluation score II; ICU: intensive care unit; NIS: non-invasive ventilatory support; HFNC: high-flow nasal cannula; CPAP: continuous positive airway pressure; NIV: non-invasive ventilation; IMV: invasive mechanical ventilation; LMR: lung recruitment maneuvers; iNO: inhaled nitric oxide; TV/PBW: tidal volume divided by predicted body weight; RR: respiratory rate; PEEP: positive end-expiratory pressure; FiO_2: inspired fraction of oxygen; P_{plat}: plateau pressure; DP: driving pressure; PaO_2: arterial pressure of oxygen; $PaCO_2$: arterial pressure of carbon dioxide; HCO^-_3: plasma bicarbonate concentrations.

Table S4. Comparison of outcomes of C-ARDS patients treated with VV-ECMO between the first and subsequent waves of COVID-19 outbreak.

OUTCOMES	FIRST WAVE* (N = 14)	SUBSEQUENT WAVES* (N = 34)	*p-value*
**Days on ECMO,** *median (IQR)*	22 (17 - 32)	14 (10 - 23)	*0.012*
**Days on IMV,** *median (IQR)*	35 (24 - 43)	23 (15 - 27)	*0.002*
**Tracheostomy,** *n (%)*	8 (57.1)	8 (23.5)	*0.025*
**LOS in ICU,** ***days,*** *median (IQR)*	38 (30 - 49)	24 (17 - 32)	*0.005*
**LOS in Hospital,** ***days,*** *median (IQR)*	44 (35 - 58)	28 (21 - 38)	*0.005*
**Mortality at day 28 post-ECMO,** *n (%)*	6 (42.9)	26 (76.5)	*0.025*
**Mortality at day 60 post-ECMO,** *n (%)*	11 (78.6)	30 (88.2)	*0.400*
**Complications:**			
**ECMO-related:**			
**Cannula thrombosis,** *n (%)*	1 (7.1)	1 (2.9)	*0.503*
**Membrane clotting,** *n (%)*	3 (21.4)	11 (32.4)	*0.449*
**Circuit change,** *n (%)*	4 (28.6)	9 (26.5)	*0.882*
**Bleeding,** *n (%)*	13 (92.9)	28 (82.4)	*0.349*
**Haemorrhagic stroke,** *n (%)*	1 (7.1)	2 (5.9)	*1.000*
**Haemorrhagic shock,** *n (%)*	1 (7.1)	1 (2.9)	*0.503*
**Superinfection,** *n (%)*	11 (78.6)	32 (94.1)	*0.140*
**Superinfection by MDR,** *n (%)*	5 (35.7)	25 (73.5)	*0.014*
**VAP,** *n (%)*	10 (71.4)	29 (85.3)	*0.416*
**BSI,** *n (%)*	8 (57.1)	10 (29.4)	*0.071*
**Septic shock,** *n (%)*	11 (78.6)	16 (47.1)	*0.045*
**Deep venous thrombosis,** *n (%)*	3 (21.4)	2 (5.9)	*0.140*
**Pulmonary thromboembolism,** *n (%)*	0	3 (8.8)	*0.546*
**Acute kidney disease,** *n (%)*	8 (57.1)	13 (38.2)	*0.230*
**Renal replacement therapy,** *n (%)*	4 (28.6)	7 (20.6)	*0.550*

* 'First wave' was defined as the pandemic time between March and August 2020; 'Subsequent waves' comprehended the time between September 2020 and December 2021.
List of abbreviations: C-ARDS: COVID-19 related acute respiratory distress syndrome; VV-ECMO: veno-venous extracorporeal membrane oxygenation; IMV: invasive mechanical ventilation; LOS: length of stay; ICU: intensive care unit; MDR: multi-drug resistant pathogens; VAP: ventilator-associated pneumoniae; BSI: bloodstream infection.

Figure S1. Timeline from the start of COVID-19-related symptoms to VV-ECMO implantation in survivor (continuous arrow) and non-survivor (dashed arrow) C-ARDS patients. Time is expressed as days, median (IQR). *List of abbreviations:* C-ARDS: COVID-19 related acute respiratory distress syndrome; VV-ECMO: veno-venous extracorporeal membrane oxygenation; IQR: interquartile range.

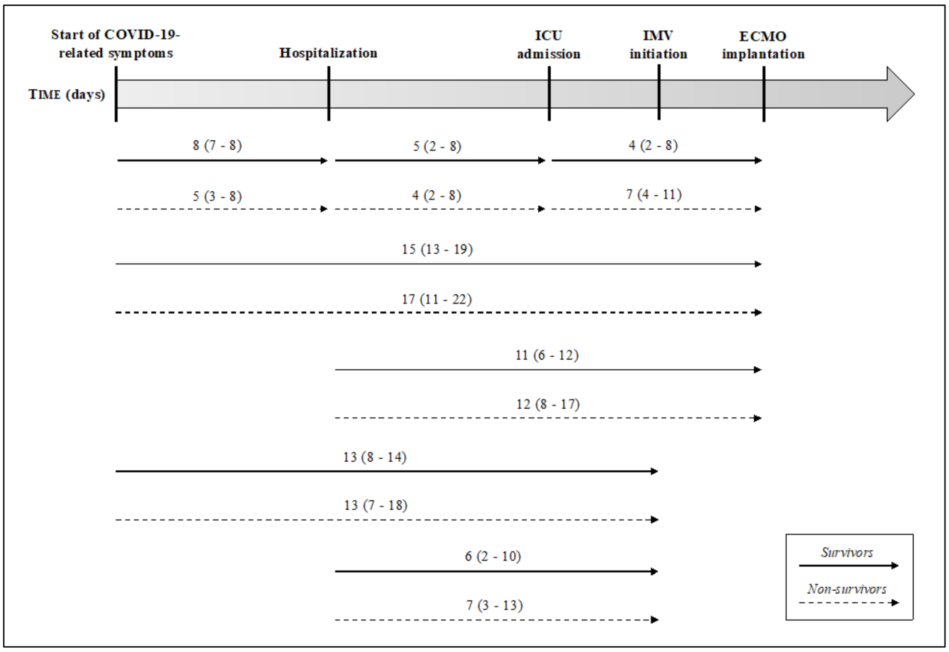

Supplement: Supplementary file 1 [file jcm-13-03545-s001.zip › jcm-2999606-supplementary.pdf]
